# Brain gray matter morphometry relates to onset age of bilingualism and theory of mind in young and older adults

**DOI:** 10.1038/s41598-023-48710-4

**Published:** 2024-02-08

**Authors:** Xiaoqian Li, Kwun Kei Ng, Joey Ju Yu Wong, Juan Helen Zhou, W. Quin Yow

**Affiliations:** 1 Humanities, Arts and Social Sciences, Singapore University of Technology and Design, Singapore, Singapore; 2 Centre for Sleep and Cognition & Centre for Translational Magnetic Resonance Research, Yong Loo Lin School of Medicine, National University of Singapore, Singapore, Singapore; 3 Department of Electrical and Computer Engineering, National University of Singapore, Singapore, Singapore

**Keywords:** Human behaviour, Cognitive ageing

## Abstract

Lifelong bilingualism may result in neural reserve against decline not only in the general cognitive domain, but also in social cognitive functioning. In this study, we show the brain structural correlates that are associated with second language age of acquisition (L2AoA) and theory of mind (the ability to reason about mental states) in normal aging. Participants were bilingual adults (46 young, 50 older) who completed a theory-of-mind task battery, a language background questionnaire, and an anatomical MRI scan to obtain cortical morphometric features (i.e., gray matter volume, thickness, and surface area). Findings indicated a theory-of-mind decline in older adults compared to young adults, controlling for education and general cognition. Importantly, earlier L2AoA and better theory-of-mind performance were associated with larger volume, higher thickness, and larger surface area in the bilateral temporal, medial temporal, superior parietal, and prefrontal brain regions. These regions are likely to be involved in mental representations, language, and cognitive control. The morphometric association with L2AoA in young and older adults were comparable, but its association with theory of mind was stronger in older adults than young adults. The results demonstrate that early bilingual acquisition may provide protective benefits to intact theory-of-mind abilities against normal age-related declines.

## Introduction

Age-related neurocognitive differences are widely documented^1^. For instance, aging negatively affects people’s performance in language production^2^, speed of processing^3^, memory and executive functioning^4,5^, as well as social cognition such as understanding the intentions, beliefs, and emotions of other people^6,7^ (see reviews in^8,9^). These changes have been attributed to concurrent age-related neural degradation in the brain, such as atrophy^10^, deficits in white matter microstructure^11^, and reduced neural efficiency^12,13^.

Beneath the typicality of age-related changes lies the substantial heterogeneity of aging, such that some people appear more resilient to adverse aging processes than their counterparts^14,15^. Lifelong experiences such as education, leisure activities, and bilingualism can modify the trajectories of the effect of aging on behavioral and neural function, acting as factors of cognitive and brain reserve, beyond genetic predispositions (see the revised Scaffolding Theory of Aging and Cognition model, STAC-r^16,17^). Cognitive reserve refers to the fact that humans can maintain or recover cognitive performance in the advent of brain injury, pathology, or aging, as a result of neuroplasticity and functional reorganization^18–21^. According to STAC-r, these lifestyle factors offer compensatory scaffolding to effect less age-related decline in cognitive function^21^ or delay to undesirable outcomes (e.g., dementia) until much later^22,23^.

Cognitive reserve is naturally related to brain reserve, its neural basis, which varies from one individual to another depending on age-related structural changes experienced by the neural system, and lifestyle factors can exert both distant (early life) and recent (ongoing) effects on it^24^. Current research typically defines brain reserve as desirable neuoroantomical properties, such as less reduction in overall brain size, more neurons and synapses (e.g., greater gray matter volume and density), and higher integrity of white matter tracts^25^. For instance, higher volume is usually associated with better cognition such as executive functioning^26^. Despite the nonlinear relationship between thickness or surface area and cognition during development^27^, by young adulthood, lower thickness, faster thinning, or smaller surface area was found to be associated with poorer outcomes, such as a higher risk for dementia^28^ or lower intelligence^27^. In this study, we considered higher values in common brain morphometric characteristics in the gray matter as an indicator of better reserve.

Owing to the need to represent and coordinate multiple languages representations, bilingualism is argued to entail cognitive processes that include constant updating of new information, switching between tasks, and inhibiting interference from task-irrelevant information. Declines in these executive control abilities^29^ have been long regarded as hallmark of neurocognitive aging^30,31^. Early acquisition of bilingualism thus has been hypothesized to enhance or preserve brain structure and function by perpetual engagement of both language-specific and domain-general cognitive processes, thereby modifying the trajectories of cognitive aging^32–35^. Indeed, previous research has reported that compared to age-matched monolinguals, bilingual older adults showed a later onset of Alzheimer’s dementia (AD)^36,37^, maintained cognitive performance against brain deterioration^38^, and had a more efficient brain structural and functional organization^22,39^. Additionally, positive effects of bilingualism in older populations appear to extend to gray matter volume (e.g., larger in volume and smaller age-related decreases) in areas related to language or cognitive control, such as the temporal pole, orbitofrontal, inferior parietal lobule, anterior cingulate, and prefrontal cortices^40,41^, as well as regions supporting other language-critical functions such as sensorimotor integration and procedural memory^42^. These findings suggest that bilingualism, as a life-course factor, can offer brain structural reserve in healthy aging, making the neural system in older bilinguals more resilient to age-related performance decline than in older monolinguals.

While research on cognitive aging and the protective effects of bilingualism has extensively explored “cold” cognitive processes such as executive control and memory, it remains unclear if other “hot” cognitive processes such as those that facilitate our social and emotional goals also benefit from bilingual experiences through enhanced brain reserve and maintenance. In particular, social cognition—the processes relevant for understanding emotions, perspectives, and mental states in order to interpret and predict the behavior of others—is essential for maintaining social relationships and social participation in older adults^43^ and contributes to their health, wellbeing, as well as cognitive functioning^44–46^. Social cognition is conceptualized as a “hot” component of cognition since it involves social or emotional aspects^47^ (but see^48^ for a further distinction between cold and hot social cognition), and previous research has shown brain differences in completing social cognition tasks versus cold cognition tasks^49,50^. Despite some mixed evidence, many studies have shown that social cognition is subject to decline in the normal aging process (see reviews^8,51^). For example, there is increasing evidence of age-related declines in the ability to attribute mental states to others (Theory of Mind; ToM), beginning from around the age of 50^7,9^.

To our knowledge, no previous work has examined whether bilingual language experience would have a positive impact in ToM aging. However, some evidence supporting this hypothesis comes from previous studies that reveal a relationship between being bilingual and ToM advantages in both children and young adults^52–55^. For example, Schroeder^56^ conducted a meta-analysis and revealed a small to medium bilingual advantage in ToM across studies that compared monolingual and bilingual preschool-aged children (mostly 3 to 5 years old) on tests of false belief. In examining monolingual and bilingual adults on the ability to consider another person’s perspectives, Navarro and Conway^53^ revealed that adult bilinguals outperformed monolinguals on their ToM task, suggesting that the effect found among children can carry over into young adulthood. Our study aimed to address this gap by examining whether such ToM enhancements from learning and using multiple languages may continue into later life through the putative mechanism of promoting brain reserve.

Recent perspectives on bilingualism have shifted away from a binary definition of bilingualism (e.g., monolingual vs. bilingual) and towards defining bilingualism as a spectrum of experiences (e.g., age of acquisition, proficiency, usage, diversity) that may selectively impact the human brain and cognition^33,57–59^. For instance, Pliatsikas’ dynamic restructuring model^60^ dissociates the effects of acquisition and consolidation of an additional language on brain structural changes, where initial exposure to a second language (L2) is proposed to primarily cause cortical gray matter changes, while continuous bilingual experience such as active usage of multiple languages is proposed to impact white matter and subcortical restructuring. In the current study, we aim to address the question of how early L2 acquisition influences cortical gray matter adaptation in relation to social cognition, specifically ToM, in both young and older bilinguals. We measure participants’ L2 age of acquisition (L2AoA), which is a reliable indicator of L2 acquisition, referring to the point at which initial exposure to L2 occurred^61^. Since the brain is remarkably malleable during early years of life, it has been suggested that early experience with a second language significantly shapes the organization and functioning of brain^62–64^. Previous morphometric studies have reported that earlier L2AoA was associated with greater gray matter density in the left inferior parietal cortex among highly proficient bilingual speakers^42,65^. Hence, early L2 acquisition appears to drive brain plasticity, which in turn may translate into efficient scaffolding processes and provide brain reserve throughout one’s life. In this study, we examined the putative neuroanatomical link between L2AoA and ToM.

What kind of neuroplasticity would early L2AoA promote, leading to subsequent preserved ToM at old age? Some researchers suggest that bilinguals’ superior ToM performance may arise from early development of sociolinguistic sensitivity, as learning different languages and communicating with people from diverse sociolinguistic background require bilinguals to regularly infer what other people know and do not know^34,66,67^. Based on this view, bilingualism may enrich brain reserve specifically associated with ToM with its higher demand for sociolinguistic abilities. Hence, one may expect the L2AoA-ToM association to manifest in selected brain regions that are implicated in mental state inferences. On the other hand, some researchers argue that bilinguals outperforming monolinguals in tasks of ToM may be attributed to domain-general cognitive enhancement because of the cognitive demand involved in language control^68–71^. As such, we would likely see a link between L2AoA and ToM in brain regions associated with language or cognitive control processes. Importantly, we view these two accounts as complementary rather than competing, where both sociolinguistic sensitivity and cognitive enhancement could contribute to the benefits bilingualism brings with it to intact ToM in older age.

In the current study, our aim was to investigate whether there are common neuroanatomical correlates of L2AoA and ToM in young and older adults. This investigation aimed to provide initial support for the neural reserve hypothesis on how bilingual experiences could influence social cognition. To capture a comprehensive set of brain morphometric characteristics (e.g.,^72^), we concurrently modelled cortical gray matter volume, thickness, and surface area. Previous aging and brain reserve studies often focused on a single cortical morphometry^26,73^. However, more recent research has shown that different morphometric features may exhibit varying sensitivity to factors such as development, aging^74,75^, disease^76,77^, and life experiences^78–80^. For instance, Claussenius-Kalman and colleagues^81^ reported that the effects of L2AoA on brain structure differed depending on the gray matter metric used. We employed partial least square correlation^82^ to maximize the covariation between these morphometric features and the two cognitive variables of interest, namely L2AoA and ToM ability. Our hypotheses were twofold: first, we expected to find a set of brain regions where larger volume, higher thickness, and/or larger surface area^42^ were associated with earlier L2AoA and better ToM performance. Second, we hypothesized that this brain-ToM association could not be solely explained by general cognitive abilities such as processing speed, memory, and executive function. Finally, since L2AoA marked a common early bilingual milestone, we expected the brain-cognition associations to be present in both age groups. However, we did not have a specific hypothesis regarding their differences^22^. It is possible that the effects might be stronger in older adults given that their cognition is often reported to be more dependent on structure compared to younger adults (e.g.,^83,84^).

The current understanding of the contribution to neural reserve due to bilingualism suggested “neuroprotective” effects in regions implicated multiple cognitive domains such as executive control, language, and memory^85^. ToM is also a hierarchical, multifaceted construct that engages both social and non-social regions^86^. In the absence of strong a priori hypotheses on region-function mapping based solely on anatomical data, we interpreted our results from brain network perspectives^87^ and considered them with respect to the corpus of published neuroimaging studies on whole-brain activation patterns (see Data Analysis, for more details).

## Methods

### Participants

Participants were 46 young adults (mean age = 21.87 years, range = 19–30; 28 females, 18 males) and 50 older adults (mean age = 63.56 years, range = 54–77; 38 females, 12 males). Table 1 presents the demographic characteristics of the sample. All participants were residents in Singapore at the point of testing. The young adults were mainly university students, and the older adults were community residing and recruited via recruitment posters in local care service centers, on social media, or through word-of-mouth. All participants received $30 gift vouchers and $80 cash remuneration for their participation.

**Table 1:** Demographic and behavioral characteristics of the sample (*N* = 96).

	Young adults (*n* = 46)	Older adults (*n* = 50)	*W*	*r*
	Mean (*SD*)	Mean (*SD*)		
Age (in years)	21.87 (2.32)	63.56 (5.56)	0.00***	> 0.99
Years of education	14.59 (1.42)	13.14 (3.39)	1516.50**	0.32
MoCA	–	27.16 (1.73)	–	–
Language characteristics				
Age of L2 acquisition (in years)	0.70 (1.74)	4.40 (2.98)	433.00***	− 0.62
Proficiency _most proficient language_ (1–10)	9.22 (0.96)	9.34 (0.86)	1073.50	− 0.07
Proficiency _2nd-most proficient language_ (1–10)	7.23 (1.56)	8.20 (1.73)	721.00**	− 0.37
Usage _most used language_ (0–1)	0.76 (0.17)	0.72 (0.17)	1333.00	0.16
Usage _2nd-most used language_ (0–1)	0.20 (0.14)	0.23 (0.15)	1050.00	− 0.09
General cognitive abilities^a^				
Processing speed (DSST)	0.76 (0.68)	− 0.70 (0.69)	2152.50***	0.87
Episodic memory (RAVLT)	0.19 (0.96)	− 0.17 (1.01)	1391.50	0.21
Working memory (2-back)	0.42 (0.68)	− 0.39 (1.09)	1690.50***	0.47
Inhibition (Stroop)	− 0.01 (0.97)	0.01 (1.04)	1134.00	− 0.01

Means and standard deviations (*SD*s) in parenthesis by age group, and results of nonparametric Mann–Whitney tests, with *r* as effect sizes and asterisks indicating levels of significance.

*MoCA* montreal cognitive assessment, *DSST* digit symbol substitution test, *RAVLT* rey auditory verbal learning test.

^a^Dependent measures for the cognitive assessments are expressed as *z*-scores, with higher scores indicative of better performance.

**p < 0.01. ***p < 0.001.

The inclusion criteria for the study were right-handed, having normal or corrected-to-normal visual acuity, normal color vision, no history of neurological or psychiatric illnesses, and at least three years of formal education. Additionally, older adults were screened for abnormal cognitive decline using the Singapore version of Montreal Cognitive Assessment (MoCA)^88^ that was validated for use in the local older population^89,90^. The maximum score a participant could receive was 30, with a recommended cutoff score of 23 for a community-based sample (following^90^); this affected one additional older adult (MoCA score = 22) who participated in the study but was not included in the final sample of 50 older adults. The mean MoCA score for the older adults was 27.16 (*SD* = 1.73), with a range of 23 to 30. Most young adults were undergraduate students who had completed pre-university education (*n* = 36), and others completed an undergraduate degree (*n* = 10). Nearly half of the older adults (*n* = 24) completed secondary school education, while others completed pre-university education (*n* = 11) or had an undergraduate (*n* = 12) or postgraduate (*n* = 3) degree, and a few completed primary school education (*n* = 2). The young adults reported having, on average, 14.59 years of education (*SD* = 1.42), which was significantly longer than that received by the older adults (*M* = 13.14 years, *SD* = 3.39), Mann–Whitney *W* = 1516.50, *p* = 0.007, *r* = 0.32.

Participants’ language background was assessed using a language background questionnaire^59^. All participants were bilingual or multilingual and all spoke English and Mandarin as two of their languages. Most young adults (*n* = 33) reported knowledge of a third language, including Hokkien (*n* = 16), Cantonese (*n* = 4), Malay (*n* = 3), Teochew (*n* = 2), Korean (*n* = 2), German (*n* = 2), Bahasa Indonesia (*n* = 1), Japanese (*n* = 1), Latin (*n* = 1), and Shanghainese (*n* = 1), and 16 of them also reported knowledge of a fourth language, including Hokkien (*n* = 5), French (*n* = 4), Japanese (*n* = 3), Malay (*n* = 3), and Teochew (*n* = 1). A majority of the older adults (*n* = 48) reported knowing English, Mandarin, and a third language, including Hokkien (*n* = 22), Cantonese (*n* = 16), Teochew (*n* = 5), Hainanese (*n* = 3), Hakka (*n* = 1), and Malay (*n* = 1), while 25 of them also reported knowledge of a fourth language, including Hokkien (*n* = 9), Malay (*n* = 8), Cantonese (*n* = 5), and Teochew (*n* = 3). However, these participants reported using predominantly two of the languages they knew (average total usage of the two most used languages was 96.49% and 94.45% in a typical week for young and older adults, respectively; see Table 1) and did not use the other one or two languages regularly. Thus, we considered all participants as active bilingual speakers in the current study.

Self-rated language proficiency (on a 10-point scale: 1 = *not proficient* to 10 = *very proficient*) also suggested that participants were highly proficient bilinguals (most proficient language: young adults, *M* = 9.22, *SD* = 0.96, older adults, *M* = 9.34, *SD* = 0.86; second most proficient language: young adults, *M* = 7.23, *SD* = 1.56, older adults, *M* = 8.20, *SD* = 1.73), despite that there were significant age differences in self-reported proficiency of the second most proficient language, *W* = 721.00, *p* = 0.002, *r* = − 0.37, but none for the most proficient language, *W* = 1073.50, *p* = 0.55, *r* = − 0.07.

Participants also reported the onset age at which they were first exposed to their second language (L2AoA) as one of our key variables. A majority of young adults acquired L2 at birth (*n* = 36) or between ages 1 and 6 (before formal schooling, *n* = 9), and one young adult reported first exposure to L2 at 8 years old. On the other hand, some older adults acquired L2 at birth (*n* = 13), many of them acquired L2 between ages 1 and 6 (*n* = 21), and some reported first exposure to L2 between 7 and 9 (*n* = 16). On average, the young adults acquired their second language at ages significantly earlier than the older adults, *W* = 433.00, *p* < 0.001, *r* = − 0.62. Furthermore, given that none of our participants reported that they stopped using their languages completely as they aged and the sample were living in a multilingual community, we considered these participants as bilinguals with continuous experience of using multiple languages since acquisition^33^.

### Procedure and measures

Each participant participated in a behavioral testing session and then an MRI scanning session conducted on separate days, with a mean interval of 20.9 days in between sessions. Note that most participants (*n* = 91) completed the MRI scan within 60 days after the first session. There were five participants (2 young adults and 3 older adults) with longer between-session intervals (72 days or more) due to scheduling restrictions during the COVID-19 pandemic; but excluding these participants did not change our results. During the behavioral testing session, all older participants were first assessed on the MoCA. All young and older adults completed four neuropsychological tasks that measure general cognitive abilities, followed by a battery of ToM tasks, and then a language background questionnaire. The order of the neuropsychological testing was as follows: Stroop task, Rey Auditory Verbal Learning Test (RAVLT), Digit Symbol Substitution Test (DSST), and 2-back task. During the MRI scanning session, participants completed functional and structural runs in the MRI scanner. While the functional MRI also involved exposing participants to socio-affective stimuli, in this report we focused on the structural data, as brain reserve typically refers to the possession of neuroanatomic resource^14^, a property that could be influenced by early life experiences^91^. All participants completed the study in English, except for 8 older adults who completed the Chinese version of the same tasks and questionnaires. The procedures were approved by the Institutional Review Board of Singapore University of Technology and Design (approval number 16-109) and National University of Singapore (approval number B-14-045) and performed in accordance with regulations in the Code of Federal Regulations Title 45 Part 46. Informed consent was obtained from all participants.

#### Theory of mind assessments

Participants’ ToM abilities were evaluated using the Theory of Mind Task Battery (ToMTB)^92^. The task battery consists of 15 questions within nine tasks, which assess ToM understanding and represent a variety in terms of content and complexity. Tasks were presented as short vignettes and arranged in ascending difficulty as follows: (a) emotion recognition task, (b) desire-based emotion task, (c) seeing leads to knowing task, (d) line-of-sight task, (e) perception-based action task, (f) first-order false belief task, (g) belief- and reality-based emotion and second-order emotion task, (h) message-desire discrepant task, and (i) second-order false belief task. There were four questions for task (a), two questions for task (d), three questions for task (g), and one question for each of the other tasks. The tasks were presented in a story-book format with colored illustrations and text, and the experimenter narrated the story and pointed to the relevant pictures while reading the story. For each test question, participants were presented with one correct response option and three plausible distractors. Except for the line-of-sight task, participants were not allowed to refer to the previous pages when answering the questions. Memory control questions were included which must be passed for credit to be given on the test questions. ToM scores were calculated as the total number of correct responses, ranging from 0 to 15 with higher scores indicating greater ToM knowledge.

#### Language background measures

Language background measures were derived from a questionnaire adapted from the language background questionnaire^59^ that was previously used to assess the language experience of bi- and multilingual adults in Singapore. Participants first listed, in order of dominance, up to four spoken languages that they have knowledge in, and for each of the languages, they reported the age at which they were first exposed to the language. Language proficiency in listening and speaking were reported on a 10-point scale where 1 is *not proficient* and 10 is *very proficient*, and then averaged to obtain one proficiency score per language. For language usage, participants first estimated the amount of time (in percent) they spent in four different social contexts (i.e., home, work, school, and others) in a typical week and then indicated how often (in proportion) they used each language in each of these contexts. The usage of the different languages across the various social contexts would add up to 1. The language characteristics of the sample were presented in Table 1.

#### General cognitive ability measures

To assess participants’ general cognitive ability, we administered four tasks that tap on different domains of general cognition: processing speed, episodic memory, working memory, and inhibition. The Digit Symbol Substitution Test (DSST) of the Wechsler Adult Intelligence Scale (WAIS-III)^93^ was used to index speed of processing. The Rey Auditory Verbal Learning Test (RAVLT)^94^ was administered, and the delayed recall score from this task was used as a measure of verbal episodic memory^95^. The computerized 2-back task^96,97^ was used to assess working memory, or the updating component of executive function. Lastly, the computerized Stroop task^84^ assessed inhibition, the ability to inhibit prepotent responses^98^. Details of the task design and score calculation can be found in Supplementary Materials. Raw scores for each of the individual cognitive assessments were transformed into *z*-scores based on the sample means and *SD*s across all participants, with higher scores reflecting better performance. Data from these tasks were collected to describe the sample characteristics (see Table 1) and used as control variables in our main analyses.

#### MRI data acquisition and pre-processing

Anatomical MR images were collected on a 3T Siemens Prisma scanner at the Centre for Translational MR Research in the National University of Singapore using a 32‐channel head coil and a T1-weighted Magnetization Prepared Rapid Gradient Recalled Echo sequence (MPRAGE; 176 sagittal slices, resolution 1 mm isotropic, repetition time = 1950 ms, echo time = 2.98 ms).

FreeSurfer 6.0^99^ was used to generate morphometric measures from the T1-weighted MRIs. Briefly, voxels corresponding to the brain was extracted using a hybrid watershed/surface deformation procedure. Images were then transformed to Talairach space and segmented into subcortical white matter and deep gray matter structures before intensity normalization. Tessellation and surface deformation of the white–gray and gray–CSF borders were employed to segment the different tissue types. Finally, images were registered to a spherical atlas that matches individual cortical folding patterns to cortical geometry across participants. Three regional features, namely gray matter volume, surface area, and thickness, were extracted from the cortex based on a 400-region parcellation atlas, which was derived from resting-state functional MRI data of a group of young adults^100^. We applied a functionally derived parcellation because structural and functional organization of the brain shows good correspondence^101^. Applying the same parcellation to both age groups ensured topographical consistencies for simultaneous analyses^102^.

## Data analysis

The first set of analyses investigated the behavioral data and assessed the effects of aging on participants’ performance on the ToM tasks. To achieve this, we conducted an ANCOVA on the ToM scores with age group (young vs. older) as a between-subject factor, with years of education and all four general cognition measures as covariates.

Next, to explore the relationship among L2AoA, ToM, and brain morphometry, we applied partial least square correlation (PLSC) to extract latent brain-cognition variables (LV)^82,103^. PLSC is a data-driven multivariate method suitable for datasets with many more variables than participants^104^. It evaluates the covariations among variables in a lower dimension latent space, i.e., the LVs^105^. Past studies have used this method to glean important insights across multiple population and neuroscientific topics^106–109^. Here, four latent variables (restricted by 2 groups × 2 cognitive measures), despite our inclusion of 1200 FreeSurfer measures, were evaluated. Each LV would reflect putative brain morphometric features best associated with L2AoA and/or ToM. Only LVs yielding reliable associations with both variables would qualify as candidates of neural reserve, and LVs captured in association with only L2AoA or ToM would not be evidence for our hypothesis. Data from the two age groups were organized as separate blocks^107^ such that these associations can vary between groups, similar to the inclusion of an interaction effect with group in typical regression models. The brain feature matrix comprised the concatenated FreeSurfer measures (subject × [morphometry × ROI]) and the cognitive measure matrix comprised L2AoA and ToM scores (subjects × measure). Both matrices were residualized with respect to gender and years of education; gray matter volume and surface area were further adjusted for estimated total intracranial volume. The correlation of the two matrices were then decomposed using singular value decomposition, yielding two LVs. The statistical significance of each LV relative to chance was evaluated based on their eigenvalue using permutation (1000 iterations, *p* < 0.05). Each LV comprised a brain salience and a cognitive salience. The brain salience was the estimated weights of each morphometric feature contributing to the LV. The behavioral salience was the strength of the relation between the multivariate brain salience and L2AoA and ToM. The robustness of the saliences was evaluated using bootstrapping (1000 iterations). For the brain salience, morphometric features with a bootstrap ratio (salience divided by bootstrapped standard deviation) ≥ 3.1, approximating a z-score of *p* < 0.001, were deemed robust for visualization and interpretation. For the cognitive salience, the procedure yielded 95% confidence intervals (CIs) of the morphometry-cognition correlations. A reliable correlation with CI excluding zero was interpreted. For visualization, brain salience was presented on an MNI brain surface template^110^.

To assist our understanding of the brain-cognition associations, we conducted two sets of multiple regression analyses. First, in additional to visual inspection of the CIs in PLSC, two multiple regressions were conducted. In each regression, L2AoA or ToM was the dependent variable, while age group, brain score, and their interaction were the primary independent variables, with gender and years of education as covariates. It is important to note that these variables were already considered in the initial PLSC analysis, so these regressions supplemented existing inferences. Second, to examine whether the identified brain salience might be contributed by individual differences in general cognition, we regressed the brain scores against four standardized cognitive scores, while controlling for gender and years of education. Note that these cognitive scores were not included in the PLSC analysis, in contrast to the first set of regressions.

To better understand the functional implications of the extracted brain salience (e.g., if the regions reflect processes related more to general cognitive ability vs. ToM-specific ability), non-thresholded brain salience was first split into topographical “maps” of gray matter volume, surface area, and thickness. Each map was then subject to separate descriptive meta-analytic decoding using NiMARE^111^. Briefly, NiMARE applied probabilistic modelling^112^ on a collection of functional MRI activation studies in Neurosynth to produce meta-analytic activation maps on 100 salient topics (https://neurosynth.org/analyses/topics/v5-topics-100/)^113^. The spatial correlation between each morphometric map and each topic map was then computed for informal reverse inference^114^, indicating what kind of cognitive functions the morphometrically covarying regions may subserve.

## Results

### Effects of age on ToM

Preliminary analyses revealed no significant gender differences nor the interaction effect between gender and age group in ToM understanding, both *p*s > 0.58, so they were combined in subsequent analyses of behavioral data. However, we kept gender as a control variable while analyzing the neuroimaging data.

Results of the ANCOVA revealed a significant main effect of age group, *F*(1, 89) = 7.08, *p* = 0.009, partial η^2^ = 0.074, when controlling for individual variances in education and general cognitive abilities. As expected, young adults (*M*_adjusted_ = 14.47, *SE* = 0.21) outperformed older adults (*M*_adjusted_ = 13.57, *SE* = 0.20) in the ToM tasks. Note that the covariates of episodic memory (delayed verbal recall from RAVLT) and working memory (accuracy in 2-back) individually contributed to performance in ToM: episodic memory: *F*(1, 89) = 5.28, *p* = 0.024, partial η^2^ = 0.056; 2-back: *F*(1, 89) = 6.06, *p* = 0.016, partial η^2^ = 0.064. The other covariates (i.e., education, processing speed, and inhibition) were not significant, *p*s > 0.23.

### Associations between ToM, L2AoA, and brain morphometry

Preliminary analyses revealed a significant negative correlation between L2AoA and ToM (τ =− 0.15, *p* = 0.032), partialling out the effects of age and bilingual usage and proficiency (i.e., language usage and proficiency of both languages). Across both young and older adults, an earlier L2AoA was associated with a better ToM score when other aspects of bilingual experience were controlled for.

**Figure 1:**
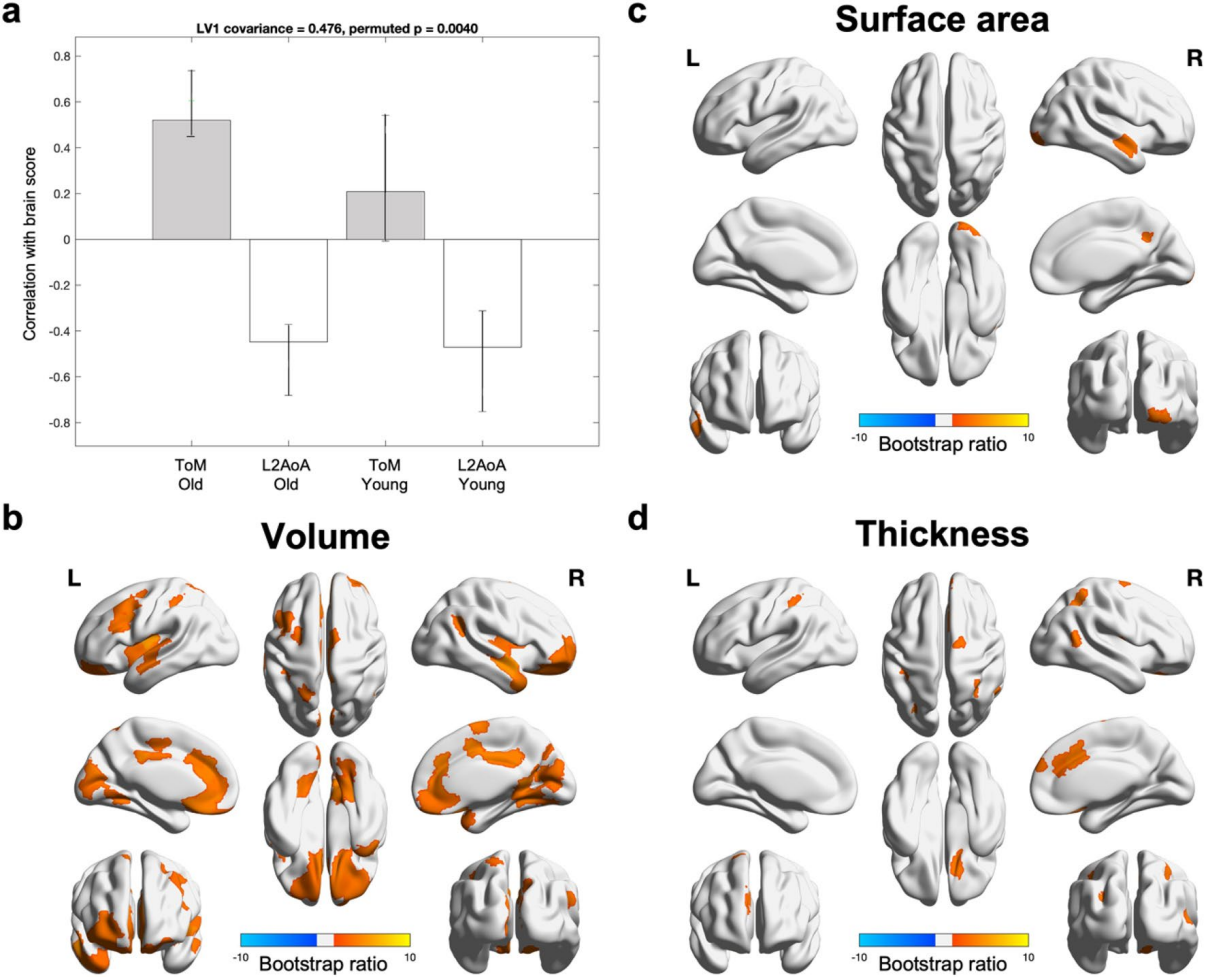
Larger gray matter volume, larger surface area, and larger thickness of multiple cortical regions were associated with earlier L2AoA and higher ToM scores. (**a**) Estimated correlation between morphometric salience and ToM (gray bars) and L2AoA (white bars) in older adults (bars 1–2) and young adults (bars 3–4). Error bars represented 95% confidence intervals. (**b**) Gray matter volume was larger in bilateral dorsolateral prefrontal, insular, medial prefrontal, medial and inferior parietal, visual, and somatomotor cortices. (**c**) Surface area was larger in the right temporal pole and medial parietal cortex. (**d**) Cortical thickness was higher in bilateral superior parietal lobules and right anterior cingulate cortex. Robust morphometric features were selected based on a bootstrap ratio of 3.1 and hot-colored.

PLSC yielded one statistically significant LV accounting for 47.6% of the covariation between the brain and cognitive metrics (*p* = 0.004). Larger gray matter volume, larger surface area, and larger thickness of multiple cortical regions were associated with earlier L2AoA (old adults: *r* = − 0.43, young adults: *r* = − 0.49) and higher ToM scores especially in the old adults (*r* = 0.55, young adults: *r* = 0.19) (Fig. 1a). This brain-ToM association was statistically stronger in the older adults than younger adults, as indicated by a significant Age Group x Brain Score interactions (*p* = 0.02), suggesting that individual differences in ToM may be more influenced by gray matter properties at older age. In contrast, the brain-L2AoA association was comparable across age groups (*p* = 0.44), suggesting early life factor influences brain morphometry similarly regardless of current age. The brain scores were also not significantly associated with the four general cognitive *z*-scores or bilingual proficiency and usage in either group (*p*s ≥ 0.1 and 0.5, respectively).

Based on a bootstrap ratio of 3.1, gray matter volume yielded the most spatially extended salience pattern, comprising temporal pole, orbitofrontal, prefrontal, posterior medial parietal, and inferior parietal cortices, most of which were bilateral (Fig. 1b). Surface area yielded the fewest robust regions and included right temporal pole and posterior medial parietal cortices (Fig. 1c). Finally, thickness yielded robust regions that comprised primarily the anterior cingulate and superior parietal cortices (Fig. 1d).

Finally, for each morphometric map, we correlated with 100 cognitive topic meta-analytic activation maps based on the Neurosynth database and identified the top 5 topics (showing the first three terms per topic below) yielding the strongest spatial correlation to infer the functions subserved by the regions expressed in that topography. The volume topography was more strongly associated with topics related to mental states (topic 71 “reasoning, mind, mental,” *r* = 0.22; topic 55 “representations, representation, abstract,” *r* = 0.20), cognitive control (topic 94 “network, control, frontoparietal,” *r* = 0.19; topic 49 “attention, attentional, task,” *r* = 0.16), and language (topic 12 “language, comprehension, sentences,” *r* = 0.18). The surface area topography was more strongly associated with language (topic 12, *r* = 0.20; topic 31 “speech, auditory, production,” *r* = 0.15; topic 23 “semantic, words, word,” *r* = 0.14) and mental states (topic 71, *r* = 0.14). The thickness topography shared more overlapping topics with volume and was more strongly associated with mental states (topic 71, *r* = 0.20; topic 55, *r* = 0.19; topic 19 “judgments, judgment, PPC,” *r* = 0.17) and cognitive control (topic 49, *r* = 0.19; topic 94, *r* = 0.17).

## Discussion

In this study, we assessed the brain structural correlates associated with bilingualism, manifested as second language age of acquisition (L2AoA), and ToM abilities in young and older bilinguals. We found a general ToM decline in older adults compared to young adults, controlling for education and general cognition. This is consistent with the current literature suggesting age-related deficits in ToM in normal aging. Importantly, we found that earlier age of bilingual acquisition and better ToM performance were associated with larger gray matter volume, higher cortical thickness, and larger surface area in distributed brain regions implicated in functions related to mental state representation, language processing, and cognitive control. This morphometric pattern associated with ToM was stronger in the older participants than younger participants, but its association with L2AoA was comparable across both age groups.

An important finding of this study is the significant association between age of second language onset and bilinguals’ brain structure, which was also related to their ToM abilities. In support of our main hypothesis, we showed that more brain reserve, as measured by gray matter metric, was associated with earlier L2AoA and better ToM performance in young and older adults. Our results provide novel evidence for how early onset age of bilingualism may modulate the developmental course of ToM abilities across the adulthood, going beyond the focus of previous work in cognitive aging on general cognitive abilities. For example, a recent study examined whether older adults’ cognition was influenced by their bilingual experience at different life stages, namely “early” (13–30 years), “middle” (30–65 years), and “late” (over 65 years)^115^. They found that participants who reported bilingualism in the early and middle life stages showed stronger correlations between regional gray matter volume in selected brain regions and cognitive performance (memory, executive functions, and language) than their monolingual peers, suggesting a possible protective role of bilingualism on cognition through neural reserve. Our results extended this potential to ToM, a key component in social cognition and everyday functioning. In line with the STAC-r framework, we show that lifestyle factors, such as early onset of bilingual experience, could establish neural reserve to protect social cognitive processes such as understanding other people’s beliefs and emotions against age-related declines.

More specifically, across three cortical gray matter morphometry, we observed larger volumes, bigger surface area, and/or higher thickness in the bilateral temporal, medial temporal, superior parietal, and prefrontal brain regions, that were generally associated with younger AoA and higher total scores in the ToM battery. In our study, the most spatially extended salience was extracted for gray matter volume. As gray matter volume is a function of cortical thickness and surface area, its dominance in robust morphometric salience may suggest that the association among cortical structure, L2AoA, and ToM is linked to a synergy of key morphometric attributes (although see^81^). Apart from visual and somatomotor cortices, the extracted morphometry featured an array of “high-level” associative regions such as the insula, orbitofrontal cortex, temporal pole, posterior medial parietal cortex, inferior parietal lobule, and medial and dorsolateral prefrontal cortices, most of which belong to the default mode, control, and salience functional networks^100^, and are implicated in the language control network^40^. Leveraging on prior knowledge through a descriptive meta-analytic procedure, this topography did in fact encompass high level cognition related to internal representations, cognitive control, and language processing that are key to ToM and social cognition in general, as discussed in detail in a recent meta-analysis of social cognition^116^. These authors also reported an activation meta-analytic cluster map corresponding to the cognitive aspect of social cognition (e.g., decoupling internal representations from the physical world, making judgments) to be primarily overlapping with the same three functional networks observed here (56% voxels with default, 9% with control, and 9% with salience).

The cortical thickness salience identified in this study encompassed mainly bilateral superior parietal lobules, medial prefrontal cortex, and some visual and somatomotor regions. Many of these regions belong to dorsal attention, ventral attention, and salience networks. Meta-analytic decoding suggested similar cognitive functions as those implicated in the volume salience. While the superior parietal cortices are not always highlighted in ToM research^116^, the impact of bilingual factors, including L2AoA, on these regions was reported before^42,64^. A recent patient study also reported impaired intention attribution ability in individuals with right superior parietal lobe tumors^117^. Cortical thickness has been shown to be malleable by skills development^118^, hence it may be effectively modulated by early L2 acquisition such that its thinning, which was reported to start as early as middle age^119^, can either be slowed down or be less detrimental to social skills.

Surface area yielded the fewest robust morphometric saliences in comparison with cortical volume or thickness, mainly in the right temporal pole and posterior medial parietal cortex, regions primarily classified as part of the default mode and a related temporoparietal network. The sparsity of this salience was similar to earlier reports^42,74^ and may be due to the higher regional specificity of surface area changes^120^. Meta-analytic decoding also showed its lower relevance with mental states and cognitive control (*r* < 0.15) compared to the other two features (*r* ~ = 0.20), but a relatively more consistent association with language and semantic processing, a “non-social” ability but highly implicated in ToM^116^, (p.309). Hämäläinen and colleagues^121^ reported that native Finnish speakers who acquired a second language earlier had more expanded cortical surface in inferior frontal and superior temporal regions than late learners. While the locations are different, they purported that such areal expansion reflected differences in phonological processing, qualitatively consistent with the implied language-related functions for our surface area topography. The prominent involvement of language processing here might be due to the heavy linguistic reliance of ToM tasks, or the fact that language acquisition and usage are inherently socially laden and motivated^122^.

The effect size of the association between this morphometric topography and L2AoA was similar in the two age groups. However, it is worth noting that between-group differences in L2AoA (earlier in young adults) and age (longer mandatory L2 experiences in older adults) may complicate its interpretation. We speculate that this finding might primarily reflect the effect of early acquisition of bilingualism (L2AoA) corresponding to the neuroplasticity to the gray matter in both groups^60^. Younger L2AoA, as seen in young adults compared to older adults, might have extended the time window for neuroplasticity (potentially resulting in more neurons), but the linear relationship might not necessarily change qualitatively between the two cohorts. This onset effect might also be distinct from the subsequent consolidation effect of increased bilingual experience^60^, which might favor older bilinguals but is also intertwined with other aspects of bilingualism such as language usage and proficiency. Future longitudinal studies that can dissociate aging and L2AoA and examine non-linear effects will provide valuable insights.

In contrast, the association between the brain scores and ToM was stronger in the older adults than young adults. The differential correlational pattern is consistent with how neural reserve may manifest: neuroplasticity is potentiated early in life and yields clearer benefits to behavioral function and health when scaffolding processes commence in later life stages^14,16^. Interestingly, visual impression suggested that the supra-threshold morphometric features have a subtle right hemispheric asymmetry. While it may be related to the right lateralization of social cognition^117,123^, brain structural reserve has also been argued to be right lateralized, especially in the frontoparietal network^124–126^. Overall, the good structure–function correspondence in our brain-cognition pattern supported our interpretation of it as a bilingualism-modulated brain structural reserve for social cognition.

In explaining how exactly bilingualism can provide cognitive and neural protection to intact ToM with aging, our results suggested that both sociolinguistic sensitivity and cognitive enhancement accounts may play a part. The sociolinguistic sensitivity account proposes that people learning and using different languages are likely to be more attuned to the challenges present in their diverse sociolinguistic environments, and therefore, early-onset bilingual experience could further shape their social understanding and potentially promote neuroplasticity related to these processes^115^. This natural embedding of social competence in language acquisition^122^ may account for the prominent inclusion of mental states processing regions in our morphometric patterns. Consistent with the cognitive enhancement accounts, our morphometric features included regions implied in cognitive control processes; however, we found no correlations between the brain scores (indicative of individual variation in brain-ToM-L2AoA association) and the general cognitive measures. Thus, our results provided a mixed picture. On one hand, it hinted that a positive effect of early L2AoA on ToM performance can occur independently of broader cognitive differences with age, consistent with past studies that failed to support the cognitive account of bilingual ToM advantage in both children and young adults^52,127^. On the other hand, it remains likely that ToM still benefited from bilingualism through its partial neural and computational overlaps with domain-general cognitive control^128–130^, which requires further investigations^71^. Equally important is our finding that L2AoA was among one of the bilingual experiences that may influence ToM and its putative structural reserve. Indeed, it has been shown that the L2 neural circuits are AoA dependent^63^, which may be attributed to, for instance, its assimilation and competition with the L1 neural circuits^122^; neurocognitive influences of L2AoA could also be independent of other bilingual aspects such as L2 proficiency, reflecting separate developmental- and experience-based pathways^131,132^. While further investigation is needed, we speculate that younger L2AoA (within the same cohort) allows the developmental and maturation trajectory of neuroplasticity to be intervened earlier, resulting in more reserve.

We noted a few limitations of the current study. First, we used a summated score to represent ToM, but ToM is likely a multidimensional construct comprising of cognitive (thoughts, beliefs, intentions) and affective (feelings, emotions) components^133^. Cognitive ToM is sometimes referred to as “cold” social cognition, while affective ToM is considered as “hot” social cognition^50^. Given past evidence suggesting that cognitive ToM might be more sensitive to age-related changes than affective ToM^129^, it would be interesting for future research to assess whether the neuroplasticity L2AoA brings with it would differ for understanding cognitive versus affective mental states. We treated a few key non-L2AoA bilingual measures as covariates, but future studies should systematically disentangle the various aspects of early-life and ongoing bilingual experiences as the benefits of bilingualism may be highly experience-dependent^22,103^. While we found earlier L2AoA to be associated with more gray matter, past studies also reported smaller gray matter in simultaneous multilinguals than in successive multilinguals^134^ (suggesting more efficient neural circuits). Despite the significant differences in aim, participant characteristics, and methodology between our study and theirs, this discrepancy requires further investigation. For example, future research could qualify L2 learning experience in terms of learning context, interaction, and duration to understand the key aspects of bilingual experience that shape brain plasticity. To account for possible age-related changes in brain topography, using cohort-specific parcellation may reveal further insights^102^. While informative, our meta-analytic decoding remains descriptive, and we did not discuss our findings in a more region- and function-specific manner. We believe that future experimental studies, in addition to anatomical investigations, will be crucial. Finally, to unambiguously demonstrate that bilingual experiences are neurocognitively protective, longitudinal studies, especially those spanning the lifespan, are essential.

In conclusion, we have shown the neural correlates between L2AoA and ToM ability in brain cortical morphometry of young and older bilinguals. Earlier age of second language onset and better ToM performance were shown to be positively associated with larger volumes, higher thickness, and/or bigger surface area in the bilateral temporal, medial temporal, superior parietal, and prefrontal brain regions. Results also indicated a stronger association of this morphometric topography with ToM performance in older adults than young adults, consistent with theories on neural reserve in cognitive aging, such as the revised scaffolding theory of aging and cognition^16^. The experience of learning and using two languages early in life could potentially be an important factor that enhances brain development in selected regions to protect age-related declines in theoy of mind.

### Supplementary Information

Supplementary Information.

## Data Availability

The datasets generated during and/or analysed during the current study are available from the corresponding authors on reasonable request.
