# Supplementary material for: Brain gray matter morphometry relates to onset age of bilingualism and theory of mind in young and older adults
Source: Sci Rep. 2024 Feb 8;14:3193. doi: 10.1038/s41598-023-48710-4 (PMC10850089; doi:10.1038/s41598-023-48710-4)
Supplement: Supplementary file 1 — Supplementary Information. [file 41598_2023_48710_MOESM1_ESM.docx]

**Brain Gray Matter Morphometry Relates to Onset Age of Bilingualism and Theory-of-Mind in Young and Older Adults**

Xiaoqian Li^1,#^, Kwun Kei Ng^2,#^, Joey Ju Yu Wong^2^, Juan Helen Zhou^2,3,4,*^, and W. Quin Yow^1,*^

^1^Humanities, Arts and Social Sciences, Singapore University of Technology and Design, Singapore

^2^Centre for Sleep and Cognition, Yong Loo Lin School of Medicine, National University of Singapore, Singapore

^3^Centre for Translational Magnetic Resonance Research, Yong Loo Lin School of Medicine, National University of Singapore, Singapore

^4^Department of Electrical and Computer Engineering, National University of Singapore, Singapore

^*^Corresponding authors: helen.zhou@nus.edu.sg; quin@sutd.edu.sg

^#^These authors contributed equally to this work.

**Supplementary Materials**

To assess participants’ general cognitive ability, we administered four tasks each taps on one specific domain of general cognition: (1) Digit Symbol Substitution Test (DSST) to index processing speed, (2) Rey Auditory Verbal Learning Test (RAVLT) to index episodic memory, (3) 2-back task to measure working memory, and (4) Stroop task to measure inhibition.

**Digit Symbol Substitution Test (DSST)**. The DSST of the Wechsler Adult Intelligence Scale (WAIS-III; Wechsler, 2000) was used to index speed of processing (following German & Hehman, 2006). It is a time-dependent substitution test in which participants copy simple graphic symbols that are paired to the digits 1–9 within 90 seconds. The DSST was administered as a paper-and-pencil test. The test score was the number of correctly entered symbols (a possible maximum of 93), with higher scores indicating better performance.

**Rey Auditory Verbal Learning Test (RAVLT)**. The task was administered as a measure of verbal episodic memory (Schmidt, 1996). Participants learned a list of 15 words (List A) over trials 1-5 and then a second list of 15 words (all different from List A) in trial 6; recall was assessed after each learning trial. Participants were asked to recall all the words from List A immediately after trial 6, and again after a 20-minute delay. Episodic memory was indexed by the delayed recall score, which was total number of words correctly recalled at long delay hence reflecting long-term verbal retention (Laillier et al., 2019).

**2-back task**. We used the 2-back task (adapted from Kane et al., 2007; Phillips et al., 2011) to assess working memory, or the updating component of executive function. This task, as well as the numeric Stroop task below, were programmed in PsychoPy (v3.2.4; Perice, 2007) and administered on a 15-inch laptop. Participants’ responses and reaction times (RTs) were recorded using a keyboard connected to the laptop. In the 2-back task, participants were presented with a sequence of numbers (1 to 9) individually in the center of the screen. Participants were instructed to press the spacebar if the number in the current trial matched the number presented two trials prior, otherwise no response was needed. Participants completed a practice block of 15 trials with feedback at the beginning of the task, and four experimental blocks (no feedback), each consisted of 18 trials, of which 6 were target trials that needed a response and 12 were lure trials that did not. Participants could take short breaks in between two blocks. Mean accuracy in the experimental blocks was used as the measure of working memory.

**Stroop task**. The numeric Stroop task (adapted from Phillips et al., 2011) assessed the ability to inhibit prepotent responses or competing representations (Friedman & Miyake, 2004). Participants were instructed to report the number of items present in the stimulus by pressing one of four keys labeled “2”, “3”, “4”, or “5” on the keyboard. Stimulus involved either a string of two to five *X*s (e.g., *XX*, *XXX*; neutral condition) or two to five repeats of the same numerals (e.g., *22*, *333*, *5555*, *44444*; congruent or incongruent condition). In the congruent condition, the number of items in the display was the same as the digit presented (e.g., *333*), while in the incongruent trials, the number of items was not the same as the digit presented (e.g., *5555*). Participants completed four experimental blocks in a fixed order: 24 neutral trials, 24 congruent trials and 24 incongruent trials (order counterbalanced across participants), followed by a mixed block of 24 congruent and 24 incongruent trials. Participants were presented with a central fixation cross for 1000 ms before being presented with the target stimulus that stayed on the screen until a response or for a maximum of 4000 ms. The measure from this task was the Stroop congruency effect, which was calculated as subtracting the mean RT of congruent trials (from the congruent and mixed blocks) from the mean RT of incongruent trials (from the incongruent and mixed blocks). For RT calculation, trials with incorrect response and outlier RTs were trimmed for each participant by excluding any trials with RT below 200 ms and those deviating by more than 2.5 *SD* of the participant’s mean across all trials (resulted in discarding 2.6% of the data). Error rates were low (mean < 3% for young and older adults) so were not further analyzed. A smaller Stroop congruency effect reflects the better ability to inhibit prepotent responses; thus, as mentioned above, the *z*-scores from this task were reversed so that higher scores indicate better inhibitory control.
